# Fibroblasts—Warriors at the Intersection of Wound Healing and Disrepair

**DOI:** 10.3390/biom13060945

**Published:** 2023-06-06

**Authors:** Jesse Roman

**Affiliations:** Department of Medicine, Division of Pulmonary, Allergy and Critical Care and The Jane & Leonard Korman Respiratory Institute, Thomas Jefferson University, Philadelphia, PA 19107, USA; jesse.roman@jefferson.edu

**Keywords:** fibroblasts, extracellular matrix, integrins, fibrosis

## Abstract

Wound healing is triggered by inflammation elicited after tissue injury. Mesenchymal cells, specifically fibroblasts, accumulate in the injured tissues, where they engage in tissue repair through the expression and assembly of extracellular matrices that provide a scaffold for cell adhesion, the re-epithelialization of tissues, the production of soluble bioactive mediators that promote cellular recruitment and differentiation, and the regulation of immune responses. If appropriately deployed, these processes promote adaptive repair, resulting in the preservation of the tissue structure and function. Conversely, the dysregulation of these processes leads to maladaptive repair or disrepair, which causes tissue destruction and a loss of organ function. Thus, fibroblasts not only serve as structural cells that maintain tissue integrity, but are key effector cells in the process of wound healing. The review will discuss the general concepts about the origins and heterogeneity of this cell population and highlight the specific fibroblast functions disrupted in human disease. Finally, the review will explore the role of fibroblasts in tissue disrepair, with special attention to the lung, the role of aging, and how alterations in the fibroblast phenotype underpin disorders characterized by pulmonary fibrosis.

## 1. Introduction

**Figure 1:**
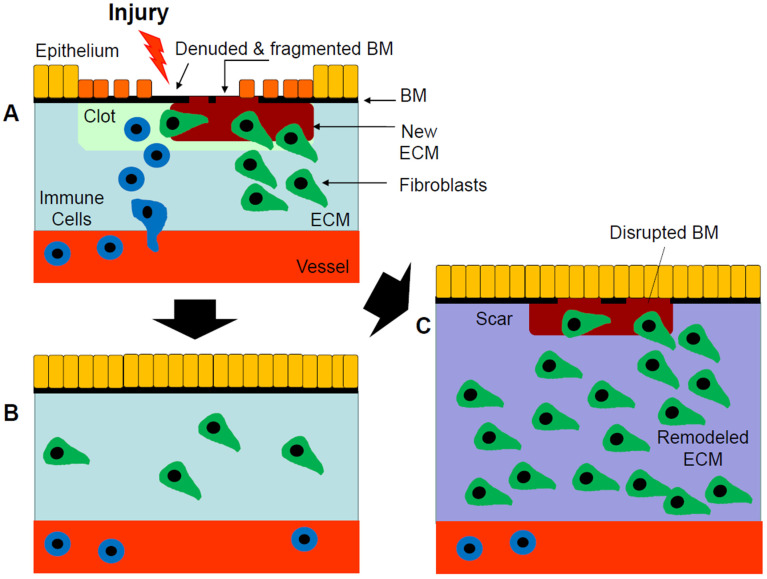
Wound healing through adaptive repair or disrepair. (**A**) In the skin or lung, injury results in a denuded and disrupted basement membrane (BM). The epithelium (yellow) becomes disrupted and activated (orange) and produces proinflammatory mediators that attract immune cells (blue), which establish inflammation to combat the injurious agent. Both epithelial and immune cells produce soluble mediators that attract and activate resident and incoming cells into the injured tissues. Fibroblasts (green) produce extracellular matrices that serve to prevent bleeding, while providing a scaffold for the re-epithelialization of denuded basement membranes. (**B**) If controlled, this wound healing response restores the original architecture, thereby preventing loss of organ function. (**C**) In contrast, if unregulated, these events lead to excessive fibroproliferation and the erratic deposition of ECMs resulting in tissue fibrosis (purple) and loss of function.

Multicellular organisms are often exposed to injurious agents capable of disrupting tissue homoeostasis. If left unattended, these injuries lead to tissue destruction and a loss of organ function. As a consequence, wound healing responses have developed to eradicate these injurious agents and repair damaged tissues. Wound healing is triggered by inflammation and characterized by the invasion and proliferation of fibroblastic cells, followed by extracellular matrix (ECM) deposition and scar formation and contraction [1]. This evolutionary conserved response is universal, and, together with inflammation, its deployment is crucial to combating infection and the noxious effects of toxins and pollutants, and to repairing tissues after trauma. If appropriately deployed, wound healing leads to adaptive repair, resulting in the preservation of the original tissue structure and function. To be successful, however, the wound healing response needs to be controlled and ultimately turned off to avoid further damage through maladaptive repair or disrepair [2] (Figure 1).

The beneficial and sometimes life-saving roles of inflammation and wound healing are most obvious in the skin and lungs, which are in continuous contact with the external environment and provide the first line of defense against injurious agents. However, the dysregulation of the host response to injury is all too frequent, resulting in human afflictions characterized by excessive inflammation, clotting, and tissue scarring [3,4]. Such clinical entities range from autoimmune disorders and thromboembolic disease to progressive tissue fibrosis [5,6,7]. A better understanding of the factors that trigger, maintain, and inactivate wound healing is essential to preventing or reversing disorders caused by tissue disrepair.

Wound healing depends on the accumulation and activation of the fibroblasts responsible for the deposition of granulation tissue and wound contraction [8]. Interestingly, tissue granulation is often considered a “late” consequence of or almost a terminal response triggered by inflammation. However, although temporal designations are useful for the study of specific biological processes, wound healing encompasses a series of processes that overlap in time and space with inflammation. These processes influence each other in ways that stimulate immune cell activation and recruitment, promote epithelial and endothelial cell homeostasis, and drive matrix deposition, followed by tissue regeneration [9,10].

This review will focus on fibroblasts and their critical function in wound healing, with special attention to the lung. It will start with a general description of the origin of fibroblasts and their heterogeneity in different tissues, followed by a discussion of the specific fibroblast functions that are considered to be important for wound healing, but may drive disrepair when unregulated. Several concepts will be emphasized throughout the presentation. First, fibroblasts are more than just structural cells responsible for maintaining tissue integrity. Second, fibroblasts derive from distinct embryonic structures and express distinct markers emphasizing the heterogeneity of this cell population. Third, activated fibroblasts are mostly responsible for ECM expression after injury, thereby representing the main effector cell responsible for tissue remodeling. Fourth, fibroblasts influence inflammation by participating in immune regulation. Finally, dysregulated fibroblast responses promote tissue disrepair, a process that underpins many human disorders.

## 2. Origin of Fibroblasts

Inflammation promotes the proliferation, activation, and migration of distinct cells into injured tissues. These events are regulated by soluble mediators that drive immune cell recruitment through the release of chemoattractants and mitogenic factors, promote the repair of injured vascular structures via the production of proangiogenic factors, and prevent excessive bleeding through the accumulation of platelets and the release of clotting factors. One of the cells recruited to these injured sites is the fibroblast, a cell derived from the embryonic mesoderm that typically resides in a semi-quiescence state within the mesenchyme or stromal tissue, where it helps to maintain the integrity of healthy tissues [11].

**Figure 2:**
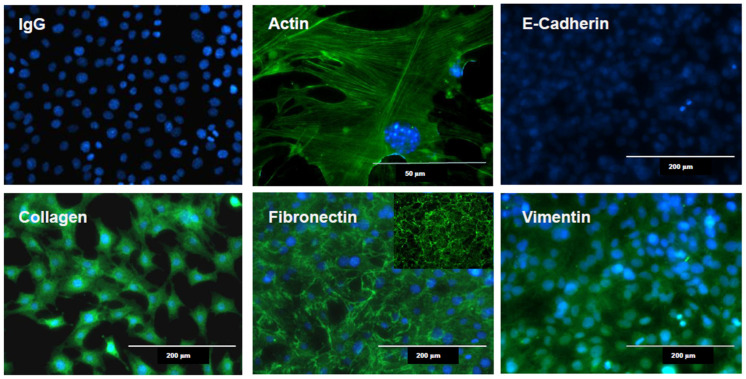
Immunofluorescence staining of cultured lung fibroblasts. When cultured on plastic or glass, fibroblasts spread out via extending actin bundles (top row, left and middle images). In contrast to epithelial cells, fibroblasts do not express E-cadherin (top row, right image), but express mesenchymal markers such as vimentin (bottom row, right image). While in culture, the cells produce collagen and fibronectin (bottom row, left and middle images; note the insert in upper left corner of the fibronectin image showing another culture with more fibronectin assembled). Note that the cells were fixed with paraformaldehyde and permeabilized with Triton X-100 (Sigma-Aldrich, St. Louis, MO, USA) prior to immunofluorescence staining.

Fibroblasts were first described by Rudolf Virchow, who defined them as “spindle cells of the connective tissue” [12]. Fibroblasts are typically defined ultrastructurally based on their stellate appearance with elongated processes. They have a prominent rough endoplasmic reticulum and Golgi apparatus consistent with high biosynthesis activity [1,13]. However, except for the identification of certain makers, such as the cytoplasmic protein vimentin or procollagen Iα2 and FSP-1, which have been used to isolate and characterize these cells, it has been difficult to determine their exact origin [14,15] (Figure 2). Recent genetic lineage tracing studies have revealed that fibroblasts originate from different embryonic tissues. In the skin of the head and neck areas, for example, fibroblasts originate from the embryonic neural crest, while fibroblasts from the embryonic paraxial mesoderm migrate to the skin covering the torso. In contrast, fibroblasts in the mesenchyme of internal organs originate from the lateral plate mesoderm [16,17]. At least three distinct embryonic lineages of fibroblasts with distinct spatial locations have been identified in the skin, each expressing different markers, including dipeptidyl peptidase 4 (DPP4, also known as CD26), delta-like non-canonical notch ligand 1 (Dlk1), and stem cells antigen 1 (Sca1) [18].

In the lungs, the mesoderm gives rise to many mesenchymal cells through stimulation by fibroblast growth factors, bone morphogenetic protein-4, Sonic Hedgehog, epidermal growth factor, retinoic acid, and transforming growth factor-β (TGFβ), among other factors. This stimulates the generation of multiple phenotypes, including airway smooth muscle cells, adventitial fibroblasts, lipofibroblasts, mesothelial cells, myofibroblasts, pericytes, vascular smooth muscle cells, fibromyocytes, and alveolar fibroblasts [19]. Each of these fibroblasts express a distinct gene expression signature, but it is uncertain whether these distinct markers always reflect a distinct phenotype or define a particular time in the evolving processes of differentiation or de-differentiation. For example, using platelet-derived growth factor receptor α (PDGFRα) as a pan-fibroblast marker, PDGFRα+ fibroblasts were found located in the alveolar niche, while PDFGRα^-^ cells were located closer to the airways and blood vessels. PDFGRa-/Axin2+ cells were considered to be the myofibrogenic progenitor populations involved in wound healing after injury [20,21].

As observed in uninjured tissues, the exact source of fibroblasts in injured tissues and the contribution of each of these phenotypes remains incompletely defined. In general, activated fibroblasts in injured tissues acquire new markers of differentiation and originate from at least three different sources. One such source are resident fibroblasts, which are present within the normal tissue mesenchyme, where they can proliferate rapidly in response to injury [22]. Activated fibroblasts or myofibroblasts can also originate from other cells. For example, epithelial cells may acquire mesenchymal features, including a loss of polarity and the expression of α-smooth muscle actin, through the process of epithelial–mesenchymal transition (EMT) mediated by SNAIL, zinc-finger E-box-binding, and basic helix–loop–helix transcription factors [23,24]. EMT has been documented in the injured kidney, lung, heart, and liver, among other organs [25,26,27,28]. Endothelial-to-mesenchymal transition has also been described [29]. Myofibroblastic cells are also found in circulation. These circulating progenitors, also termed fibrocytes, express hematogenous cell markers (e.g., CD34+) and procollagen type I, and their presence has prognostic implications in certain disorders [30,31,32]. Of note, the contribution of these fibroblast subtypes to the overall fibroblast population present in injured tissues differs depending on the site. In models of unilateral ureteric obstruction, studies using bone marrow chimeras and transgenic reporter mice revealed that resident fibroblasts, EMT, and circulating progenitors contributed to 52%, 38%, and 9% of the fibroblasts, respectively [33]. A similar fibroblast population heterogeneity has been described in other organs [34].

In summary, fibroblasts represent a heterogenous population of mesenchymal cells with distinct origins. Their heterogeneity is best highlighted by the distinct surface markers they carry, but whether these markers define a distinct cell phenotype or reflect a window into the evolving processes of differentiation or maturation is not always clear. Most importantly, it appears that these distinct fibroblast subtypes reflect cells with distinct functions. Further work using cell lineage technology aligned with functional studies will be needed to better define these populations and their functions in health and disease in different tissues.

## 3. Activated Fibroblasts

**Figure 3:**
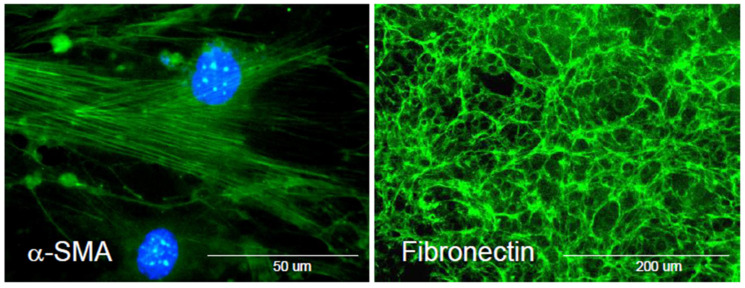
Immunofluorescence staining of activated fibroblasts and fibronectin production. (Left Image), Activated fibroblasts express bundles of α-smooth muscle actin and other cytoskeletal proteins. (Right Image), Activated fibroblasts produce fibronectin, a matrix glycoprotein implicated in tissue injury and repair. The cells were fixed in paraformaldehyde and permeabilized with Triton X-100.

Fibroblasts typically reside within mesenchymal tissues in a semi-quiescence state. During wound healing, however, fibroblasts acquire activation markers such as fibroblast activation protein (FAP), a type II integral membrane glycoprotein with dipeptidyl-peptidase and type I collagenase activity, and experience a loss of the Thy-1 glycoprotein (CD90) [35,36]. Several other markers have been used to further define these populations in the skin [37]. The most well-known sign of fibroblast activation is the appearance of myofibroblasts, which are cells manifesting features of both fibroblasts and smooth muscle cells, such as the longitudinal cytoplasmic bundles of microfilaments that are typically seen in smooth muscle cells (Figure 3) [38]. Thus, cytoskeletal protein differentiation markers are used to identify activated fibroblasts. Of these markers, the most well-known is α-smooth muscle actin. This is one of the six actin isoforms found in eukaryotic cells, with β and γ chains being present in all the cells, while α isoforms are more tissue-specific [39].

As observed for quiescence fibroblasts, not all activated fibroblasts are alike. Early studies demonstrated that myofibroblasts differ in the smooth muscle proteins they express. For example, while α-smooth muscle actin appears to be universal, desmin and smooth muscle myosin are not [40]. In fact, four different myofibroblast phenotypes have been identified in skin wounds. Independent of their phenotype, the number of these myofibroblasts has been shown to be proportional to the rate of wound contraction [41].

The different phenotypes of myofibroblasts observed likely reflect their different origins. Although data point to myofibroblasts being derived from the activation of resident fibroblasts, evidence has surfaced that other mesenchymal cells such as pericytes, stellate cells, and perisinusoidal cells contribute to this population in the liver [42]. In the kidneys, myofibroblasts originate from distinct subpopulations of pericytes and fibroblasts [43]. In general, the origins of these cells may depend on the nature of the injury, as experimental models of liver fibrosis caused by carbon tetrachloride show fibroblasts derived from hepatic stellate cells, while perisinusoidal and other fibroblasts in portal regions become myofibroblasts after bile duct ligation [44]. In mice, somatic progenitor cells expressing the transcription factor Engrailed-1 (En1) produce a fibroblastic cell lineage that promotes most of the scarring that occurs in injured skin [45]. In the ventral skin, however, progenitors expressing paired related homeobox 1 (Prrx1) give rise to fibroblasts responsible for fibrotic responses in the chest and belly skin [46]. Other investigators have identified differential patterns of skin fibroblast migration based on the expression levels of Wnt genes [47].

Together, studies of the activated fibroblasts accumulating in injured tissues have emphasized the heterogeneity of these cells and their diverse origins. Furthermore, they have pointed to the plasticity of these cells, which might lead to the accumulation of distinct fibroblast populations with varying biosynthetic activity and functions, depending on the nature of the injury and organs involved.

## 4. Soluble Modulators of Fibroblast Function

Injured cells produce soluble mediators that stimulate fibroblast activation into myofibroblasts and influence fibroblast functions. Examples of such factors include macrophage-derived PDGF and Fibroblast Growth Factor-2, which stimulate fibroblast proliferation [48,49,50]. These mediators bind receptors on the surface of fibroblasts, where they trigger downstream protein kinase activation and other intracellular signals that influence cytoskeletal organization and differential gene expression. These events are necessary to effect adequate wound healing. However, an excessive production of the factors capable of the uncontrolled activation of fibroblasts may lead to tissue fibrosis.

Perhaps the best known growth factor capable of promoting fibroblast activation is TGFβ. TGFβ is a growth factor and cytokine involved in paracrine signaling that is produced by the macrophages, epithelial cells, and fibroblasts in many organs [51]. It belongs to a family of mammalian TGFβ isoforms (TGFβ1, 2, 3) that includes activin/inhibin and bone morphogenetic proteins. TGFβ1 is secreted with a latency-associated pro-peptide or LAP, but can be activated by the cleavage of the LAP by matrix metalloproteinases or oxidative stress, among other mechanisms [52]. Cells can also induce TGFβ activation through cell surface integrins (e.g., β6) [53]. Once activated, TGFβ binds the TGFβR1 or TGFβR2 receptors on the surface of fibroblasts, stimulates their activation and proliferation, and induces their production of ECMs via the activation of transcription factors termed Smads, among other signals [54]. The induction of ECM expression can also occur indirectly, via the TGFβ induction of connective tissue growth factor (CTGF), another potent stimulus of myofibroblast differentiation and ECM production [55]. Excessive TGFβ production may lead to fibrosis in the liver, kidneys, and heart [44,56,57]. In the lungs, adenovirus-overexpressing TGFβ induces tissue fibrosis [58], while this fibrosis is inhibited by anti-TGFβ1 neutralizing antibodies or genetic manipulation disabling the TGFβ receptors or their signals [59]. Because of its prominent role in fibrogenesis in many organs, TGFβ is considered a “master” regulator of fibrogenic responses.

## 5. Fibroblasts and Wound Healing

**Figure 4:**
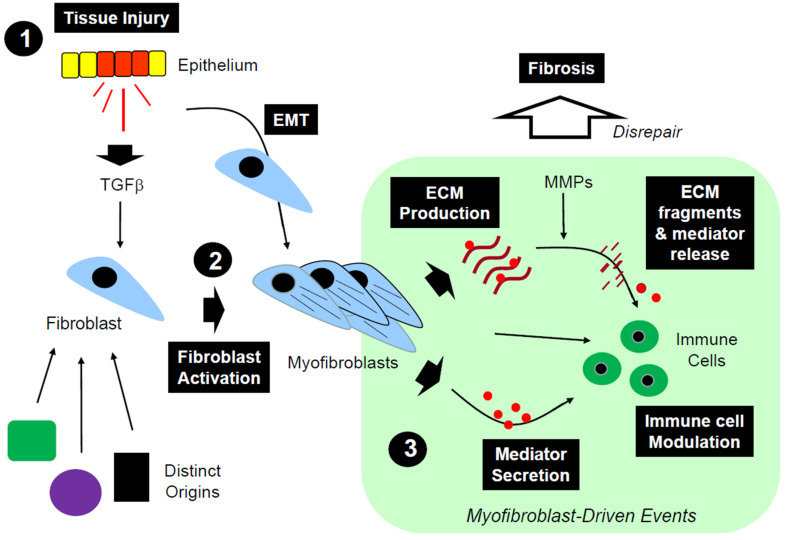
Mechanisms of wound healing and tissue disrepair (Step 1), injured epithelial cells produce soluble mediators including TGFβ that activate fibroblasts and induce epithelial–mesenchymal transformation (EMT), resulting in the accumulation of fibroblasts (Step 2). Activated resident fibroblasts, epithelium-derived fibroblasts and circulating fibrocytes accumulate in injured tissues where they deposit ECMs and produce soluble mediators, and influence immune cell function (Step 3). ECM degradation via MMPs helps release ECM fragments and bioactive mediators that affect immune cell functions. If unregulated, these events cause excessive deposition of ECMs, resulting in progressive fibrosis.

After an injury, fibroblasts accumulate and engage in the process of wound healing. This wound healing depends on at least three important processes: (1) the deposition of ECMs, (2) the production of soluble bioactive mediators, and (3) immune cell regulation (Figure 4).

## 6. Fibroblast-Derived ECMs in Health and Disease

ECMs are produced by epithelial and endothelial cells, as well as mesenchymal cells such as smooth muscle cells. However, the fibroblast is the main cell type in mesenchymal tissues that produces the ECM core components such as fibronectin and collagens. The ECM plays an important role in maintaining tissue homeostasis and is remodeled to adapt to the mechanical, biochemical, and structural changes that occur throughout the lifespan of an animal. These changes influence cell differentiation and tissue organization [60]. The remodeling of the ECM is regulated through diverse mechanisms, including ECM protein synthesis, post-translational modifications, deposition, and degradation. These events control the composition of the ECM, its dynamic organization into tri-dimensional structures, and its biochemical properties. Collagen production represents a good example of the complexity of these events, as it includes collagen synthesis through a multistep process, beginning with the transcription of collagen genes, the splicing of the pre-RNA resulting in procollagen, the formation of pre-pro-polypeptide chains that travel through the endoplasmic reticulum for post-translational processing into pro-collagen, the removal of the N-terminal signal peptide, the addition of hydroxyl groups to lysine and proline amino acid residues and their glycosylation, and the assembly of pro-collagen chains into triple helixes through zipper-like folding [61]. Procollagen is assembled into secretory vesicles. Once secreted, collagen peptidases cleave the ends of pro-collagen, converting the molecule into tropo-collagen, which forms collagen fibrils. This process for collagen, while complex, is not unique and explains how alterations in any of these steps in a given tissue might alter the expression and deposition of the ECM components in ways that affect the content and relative composition of the ECM, thereby affecting cells differentially.

In healthy states, the mesenchyme contains few mesenchymal cells (including fibroblasts) surrounded by a network of intertwined ECMs. These ECMs are arranged into two types of compartments: the interstitial matrix and basement membranes [62]. The interstitial matrix is a loose matrix meshwork that interconnects cellular structures and maintains the three-dimensional organization and biochemical characteristics of the lung. Basement membranes, on the other hand, are arranged into thin, specialized ECM layers located beneath epithelial and endothelial cell sheets. These basement membranes separate distinct cellular structures from one another, thereby allowing for the development of specialized and functional structures [63].

Advances in tissue isolation and processing, as well as newer identification techniques, have proven invaluable in defining the ECM composition of distinct organs. Recently, for example, the composition of the ECMs in mouse and human lungs has been characterized by isolating the matrix after a removal of the cellular material (decellularized scaffold), followed by the proteinase digestion of the matrix and an analysis of its components using mass spectrometry. The collection of the proteins, glycoproteins, proteoglycans, and associated modifying proteins identified by this type of analysis has been termed the “matrisome” [64]. In one study, this approach allowed for the identification of 94 proteins in the normal lung “matrisome” (61 core ECM proteins such as collagens, fibrillin-1, fibronectin, elastin, vitronectin, laminins, decorin, and tenascin) and 33 ECM-related proteins (e.g., protein-glutamine g-glutamyltransferase 2, α1 antichymotrypsin, and surfactant-associated protein A1) [65]. Further work has revealed hundreds of different ECM proteins and modifying enzymes, with some novel ECM proteins becoming more evident during injury, such as Emilin-2 and collagen XXVIII [66,67]. Emilin-2 was localized to perivascular and peribronchial regions, but appeared in the alveolar regions near the myofibroblasts in bleomycin-injured lungs. Collagen-XXVIII was localized around vessels, airways, and alveoli, consistent with it being a basement membrane protein in uninjured lungs, but its expression increased in fibrotic foci after bleomycin injury. The authors concluded that, like fibronectin, these may represent the provisional ECM proteins involved in tissue repair after injury. A proteomic analysis also identified different ECM components among mouse and human tissues [66,67]. The composition and properties of the ECM are also heterogeneous among the different regions within the lung (i.e., bronchi, bronchioles, alveoli, and vasculature) and among physiological states (i.e., fetal, adult, aged, and injured) [67,68].

The above described homeostatic state in healthy tissues is greatly disrupted after tissue injury, leading to alterations in the relative content and composition of the ECM [67]. One of the early ECMs produced by activated fibroblasts during wound healing is fibronectin, a matrix glycoprotein that assembles with fibrinogen to promote coagulation [69]. While fibronectin fragments serve as chemoattractants to immune cells [70], the deposition of fibronectin also promotes the accumulation and crosslinking of fibrillar collagens, thereby aiding the deposition and assembly of other ECM components [71]. The early deposition of ECMs such as fibronectin provides a scaffold for immune cell adhesion and facilitates the migration of epithelial cells to denuded basement membranes, thereby promoting tissue regeneration [72].

The other ECMs expressed early after injury are fibrillar collagens (e.g., collagens types I and III). Like fibronectin, their soluble fragments are chemoattractants to neutrophils and monocytes, while insoluble matrices promote migration towards injured tissues through the detection of ECM concentration gradients, termed haptotaxis [73]. The anionic, nonsulfated glycosaminoglycan hyaluronan and the proteoglycan decorin are also highly expressed in injured tissues and are known to affect cell functions [74,75].

### 6.1. ECM Recognition by Integrins

**Figure 5:**
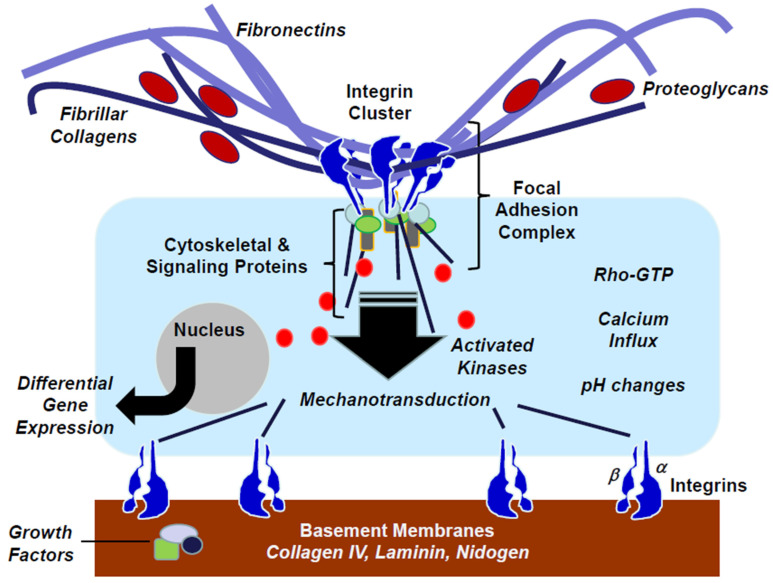
ECM–integrin binding triggers signal transduction. Cells express surface heterodimeric integrin receptors that bind ECMs located extracellularly in the loose mesenchyme or organized into basement membranes. Upon binding, integrins cluster at the surface and attract cytoskeletal and signaling molecules to the focal adhesion complex. This signaling apparatus elicits calcium influx, protein kinase activation, and other signals that promote differential gene transcription. The interaction between ECMs, integrins, and cytoskeletal structures induces strain and also promotes signaling through mechanotransduction. Adapted from George G, Ramirez MI, Roman J. Lung Mesenchyme. Murray & Nadel’s Textbook of Respiratory Medicine—7th Edition. Publisher: Elsevier. Phhiladelphia, PA, USA. Eds: Broaddus, Ernst, King, Lazarus, Sarmiento, Schnapp, Stapleton, and Gotway. 2021 [77].

ECMs not only provide support for cell adhesion and tissue integrity, but also influence cellular functions, which is possible because cells express on their surface proteins, such as integrins, capable of matrix recognition. Integrins are heterodimeric cell surface proteins that are members of a large multi-gene family of receptors involved in cell–cell and cell–ECM binding (Figure 5) [76]. Mammals express 18 α subunits and 8 β subunits capable of forming at least 24 receptors through the non-covalent binding of one α to one β subunit. These β subunits could be shared amongst integrins, but the α subunits typically confer a ligand-binding specificity.

ECMs bind to the extracellular domain of integrins through an evolutionarily conserved arginine–glycine–aspartic acid (RGD) amino acid sequence [78]. This motif is found in fibronectin, vitronectin, osteopontin, collagens, thrombospondins, fibrinogen, fibulin-5, and other ECMs. ECM–integrin binding triggers conformational changes in the receptor’s intracellular domain that attract cytoskeletal and signaling components. These ECM–integrin–cytoskeletal units are termed focal adhesion complexes (FACs) [79] and attract intracellular signaling molecules that assemble and elicit calcium fluxes, pH changes, and the activation of protein kinases, among other signals [79,80]. These signals promote differential gene expression in what is termed “outside-in” signaling. Conversely, intracellular signals may affect integrin activation, thereby affecting ECM binding through “inside-out” signaling. These interactions provide a bidirectional integration of the ECM with the intracellular signaling machinery.

ECM–integrin–cytoskeletal interactions also transfer tensile strength into cells. Not unexpectedly, this tension is dependent on the ECM composition and density, its biochemical properties, and its spatial presentation [81]. Atomic force microscopy has confirmed that ECM stiffness occurs in fibrotic disorders in humans and experimental animals, and these changes have implications for cellular signaling [82]. Fibroblasts are known to align along ECM fibers and invade the tissues in areas of reduced matrix rigidity [83].

### 6.2. Transitional Remodeling

Essentially every form of injury triggers alterations in ECM synthesis, deposition, and turnover, which result in overt architectural changes that affect organ function. In the lungs, this is highlighted by the destruction of lung parenchyma observed in emphysema, airway remodeling observed in asthma, vascular remodeling demonstrated in pulmonary vascular disease, cavity formation in tuberculosis, and fibrosis seen in the setting of idiopathic pulmonary fibrosis [84,85]. However, tissue remodeling can occur without overt changes in the tissue architecture. This process has been termed transitional remodeling and is characterized by subtle alterations in the relative composition of the ECM without resulting in microscopic or macroscopic changes in the tissue architecture [86]; in fact, this tissue remodeling can only be detected via biochemical studies, an evaluation of the mRNA expression, or immunohistochemistry. The exact impact of transitional remodeling on organ function is unknown, but it is not hard to imagine that such changes affect cellular adhesion, migration, and differentiation, among other processes, considering that cells have the capability of recognizing distinct ECM components via integrin receptors. Chronic nicotine exposure, alcohol use, and aging have been shown to trigger this transitional remodeling in the lungs, characterized by increased expressions of fibronectin, fibrillar collagens, plasminogen activator inhibitor-1, matrix metalloproteinases, and TGFβ1 [87,88,89]. Although the role of transitional remodeling remains unclear, it has been speculated that it may prime organs and increase their susceptibility to disrepair after injury, but this requires confirmation.

## 7. Fibroblast and Bioactive Molecules

In addition to providing a scaffold for cell adhesion and migration, ECMs serve as reservoirs for growth factors and other mediators. Fibroblast growth factors, vascular endothelial growth factor, hepatocyte growth factor, and insulin-like growth factor, among others, can be found embedded within the ECM, where they may associate with matrix proteins or heparan sulfate [90]. Newly secreted latent LAP-bound TGFβ1, as another example, binds to latent TGFβ-binding proteins (LTBPs) that secure it to the ECM [53].

The release of these mediators is dependent on the degradation of the ECM via proteinases belonging to the families of matrix metalloproteinases (MMPs), adamalysins, and cysteine and serine proteases [91]. The MMP family includes over 30 zinc-dependent endopeptidases produced by fibroblasts, tissue macrophages, epithelial cells, and other cells. MMPs are secreted with their inhibitors TIMPs 1–3 (for Tissue Inhibitor of MMP) at a 1:1 ratio and their activity depends on the relative concentration and spatial localization of these inhibitors. In addition to MMPs, fibroblasts release lysyl oxidase, which also helps to degrade ECMs, thereby facilitating the invasion of cells into injured tissues and affecting the tissue rigidity and porosity [92].

In addition to the release of soluble mediators via ECM remodeling, fibroblasts themselves secrete and/or activate bioactive factors that may affect wound healing, such as TGFβ. The LAP portion of newly released latent TGFβ contains an RGD consensus site for binding certain cell surface integrins (e.g., αvβ6 and αvβ1). After this binding, the integrins activate latent TGFβ1 through the release of the LTBP via mechanical strain [53].

In short, in addition to their exaggerated production of ECMs, fibroblasts secrete bioactive mediators or aid in their release by remodeling the underlying ECM with the help of MMPs. In doing so, the released bioactive molecules act in paracrine ways to influence the functions of other cells.

## 8. Fibroblasts and Immune Regulation

Multiple studies have documented that fibroblasts are also involved in immune regulation. Antigen-presenting cells such as dendritic cells migrate through the mesenchyme into draining lymph nodes after processing new antigens. There, they prime naïve T cells and stimulate immune responses depending on the antigen presented. These events are partly modulated by the ECM in which these cells are immersed. In fact, it is now recognized that fibroblast-derived ECMs help to establish a microenvironment that influences immune surveillance [93]. Cutaneous Tregs express integrin α2 (Itga2), which forms a receptor for collagens and regulates T cell migration and proliferation [94]. Immune cells can also interact with hyaluronan, a glycosaminoglycan, via CD44, which enhances cell migration [95].

Similar to professional antigen-presenting cells such as dendritic cells and macrophages, mesenchymal cells can also present antigens to T cells. This has been shown for endothelial cells and fibroblasts, among other cells [96]. In cancer, MHCII-expressing cancer-associated fibroblasts can promote immunosuppression via activating CD4 T cells [97]. Indirectly, dermal fibroblasts induce the maturation of dendritic cells [98] and the activation of macrophages. Activated fibroblasts also express higher levels of ICAM1, which enhances their interactions with dendritic cells [99].

Wound fibroblasts also secrete proinflammatory cytokines such as tumor necrosis factor-α and interleukins, as well as C-C and C-X-C chemokines, which serve to recruit neutrophils, monocytes, and macrophages to the affected sites [100].

## 9. Fibroblasts and Lung Disrepair

Having described the general concepts about the origin, heterogeneity, and general functions of fibroblasts in wound healing, and how these cells may drive tissue remodeling and disrepair, we now turn our attention to the lung and lung disorders that provide dramatic examples of how fibroblast dysregulation may drive disease development. Perhaps the disease that most reflects the impact of fibroblast dysregulation in the lungs is idiopathic pulmonary fibrosis (IPF), a condition characterized by uncontrolled fibroblast proliferation and progressive fibrosis, which lead to an irreversible loss of lung function and respiratory failure [101,102]. Although the exact mechanisms that initiate IPF and drive its progression remain incompletely elucidated, there is a general consensus that the disease is triggered by epithelial cell injury. In turn, injured epithelial cells release mediators that drive fibroblast accumulation, activation, and their secretion of bioactive mediators and ECMs, which promote uncontrolled tissue remodeling (Figure 4). These events, in the appropriate genetic background [103], lead to the development of IPF.

The above proposed paradigm places the epithelium at the center of IPF development and considers fibroblast proliferation as a secondary response. Whether fibroblasts are simply effector cells driven by epithelial-derived factors remains unclear, but several observations have suggested that, at the very least, fibroblasts have a prominent role in disease progression and represent an attractive target for intervention. Reaching this conclusion, however, has taken significant effort, because despite the many instructive experiments performed on in vitro and ex vivo models, studies exploring the exact roles of fibroblasts and fibroblast-derived ECMs in vivo have been hindered by the lack of animals that perfectly resemble the human condition, the lack of specific tools for intervention, and the fact that genetically engineered animals with mutations designed to knockout the expressions of individual ECM components and ECM-binding integrins are embryonically lethal [104,105].

**Figure 6:**
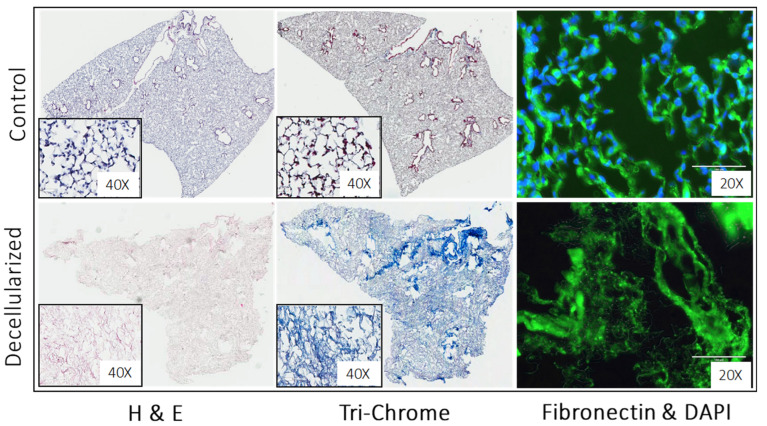
Decellularized murine lungs. (Upper row): Control lungs stained for hematoxylin and eosin stain (left image; hematoxyline stains cell nuclei in ‘purplish blue’ and eosin stains ECM and cytoplasm ‘pink’), trichrome stain (middle image; stains collagens in ‘blue’), and immunofluorescence stains for nuclei (stained with DAPI in ‘blue’) and fibronectin (right image; stained in green using anti-fibronectin antibodies). (Bottom row): After harvesting, lungs were treated with enzymes that degrade cells and DNA, thereby leaving behind only decellularized ECM (stained for fibronectin in green fluorescence) that retains the general architecture of the lung.

Despite the above limitations, three important observations greatly extended our understanding of the roles of fibroblasts and ECMs in vivo in the lungs. The first set of studies were performed with tissue sections of decellularized control and diseased tissues obtained from healthy lungs and lungs from subjects with IPF, which retain significant portions of the lung architecture after decellularization (Figure 6) [65]. When normal or inactive fibroblasts were cultured atop of the IPF lung sections, they showed an increased expression of α-smooth muscle actin when compared to the cells cultured atop of the healthy lung sections, thereby emphasizing the importance of the lung ECM in fibroblast activation after injury. Similar experiments evaluating the transcriptomes and translatomes of primary fibroblasts cultured on decellularized lung ECMs from IPF or control patients revealed that the origin of the ECM had a greater impact on the gene expression than the origin of the cells did, and that differences in the translational control were more prominent than alterations in the transcriptional regulation [106]. These observations led the authors to conclude that activated fibroblasts may pathologically remodel the ECM via a positive loop between the fibroblasts and aberrant ECM. In other words, once remodeled, the ECM may drive the further activation of fibroblasts in ways that perpetuate disrepair, independent of the fate of the initial injury.

**Figure 7:**
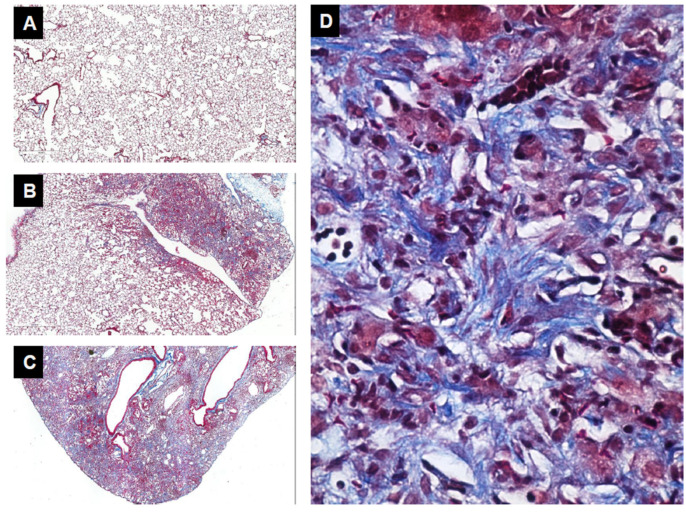
Bleomycin-induced lung injury and fibrosis. Bleomycin-induced lung injury is a well characterized model of lung fibrosis. After its administration to a normal lung (**A**), bleomycin induces epithelial cell injury and inflammation (**B**) (days 1–7), followed by the accumulation of fibroblasts and the extensive deposition of ECMs, including collagen identified in blue by trichrome staining (**C**). Upon higher magnification, note the erratic alignment of fibroblasts embedded in a newly deposited collagen matrix (**D**).

The second set of experiments was performed on animals with mutations, preventing the expression of fibronectin EDA. Fibronectin EDA, for extra domain A, is one of the many splicing variants of a single fibronectin gene. While knockout mutations in the fibronectin gene result in embryonic lethality, animals deficient in fibronectin EDA live. When exposed to bleomycin, a well-characterized model of lung injury and fibrosis, wild-type rodents develop pulmonary fibrosis (Figure 7), but fibronectin EDA knockouts are protected [107], representing one of the first studies directly testing the role of an ECM component in lung fibrosis in vivo and suggesting that the excess production of fibronectin EDA may drive disrepair. Yet, another piece of evidence that points to fibroblasts as important drivers of disease in IPF relates to the recently developed, so-called antifibrotic drugs, nintedanib and pirfenidone, which are known to target fibroblast responses to TGFβ1, among other mechanisms of action [108,109].

Considering the data implicating fibroblasts and fibroblast-derived ECMs as major drivers of disease in the lungs, one must consider the possibility that phenotypic changes in fibroblasts might increase their susceptibility to disrepair in certain individuals. Above, we described that genetics represent an important host factor in the development of IPF. However, most of the genetic mutations described, such as mutations in MUC5B and surfactant proteins, affect the epithelial cells rather than fibroblasts [110]. More recent observations appear to shed light onto how fibroblast phenotypic changes may render the host susceptible to lung disrepair after injury. These observations relate to the process of aging, which will be discussed next.

## 10. The Aging Fibroblast

Many disorders characterized by fibroproliferation and excess tissue remodeling occur in advanced age. This is consistent with studies showing that aging organs are less capable of mounting successful repair responses in humans and experimental animals, indicating their susceptibility to disrepair after injury [89,111,112]. Several processes have been implicated in age-related susceptibility to tissue disrepair, including epithelial cell dysfunction, endoplasmic reticulum stress, mitochondrial dysfunction, and stem cell exhaustion [113,114,115]. Aging is also associated with oxidative stress, but the mechanisms responsible for this occurrence, the cells driving this process, and how these events contribute to tissue disrepair after injury in the aging lung remain poorly elucidated [116]. Of interest is the fact that aging organs also show alterations in their ECMs. Aging lungs, for example, show physiological enlargements in their airspaces, resulting in the so-called “emphysema of aging”. This phenotype is associated with significant alterations in the relative ECM content, with decreased elastic fibers, increased collagen type III, and changes in proteoglycans, among other changes [117,118]. In mice, aging lungs show increased expressions of fibronectin, collagen I, MMPs, and the pro-fibrotic factor TGFβ1 [89]. These changes do not lead to overt structural or functional impairment and this represents a form of transitional remodeling. Nevertheless, as stated earlier, these changes might render the host susceptible to tissue disrepair after injury.

Since fibroblasts are major producers of ECMs, one would expect to detect alterations in the fibroblast phenotypes of aging tissues. Using distinct aging cell culture models, investigators have noted alterations in the expression levels of many tissue remodeling genes in human skin fibroblasts [119]. Similarly, primary lung fibroblasts harvested from old mice show increased expressions of pro-fibrotic and senescence genes when compared to fibroblasts obtained from the lungs of young animals [120]. Exactly why this occurs is unclear, but aging leads to increased transcriptional noise, suggesting epigenetic dysregulation [121]. Oxidative stress has also been implicated. One form of oxidative stress occurring in aging relates to the oxidation of the redox potential (Eh) for the thiol disulfide couple cysteine (Cys) and cystine (CySS), known as Eh Cys/CyS [122]. It has been postulated that the dysregulation of this process might drive lung tissue disrepair after injury and disrepair in aging organs [123].

Consistent with the above, murine primary lung fibroblasts cultured in an oxidized environment (oxidized Eh Cys/CySS) show increased proliferations and enhanced expressions of pro-fibrotic growth factors (i.e., TGFβ), myoblastic markers of differentiation (i.e., α-smooth muscle actin), and ECMs (e.g., fibronectin) [124]. Thus, Eh Cys/CySS oxidation promotes a pro-fibrotic phenotype in primary lung fibroblasts. Interestingly, fibroblasts harvested from the lungs of old animals manifested this phenotype and produced a more oxidized extracellular environment than those from young mice, at around 40 mV more oxidized [125]. Importantly, old fibroblasts displayed an impaired capacity to recover from an oxidative challenge. Thus, lung fibroblasts are not only affected by Eh Cys/CySS, but they influence it, a process that is deficient in aging cells.

**Figure 8:**
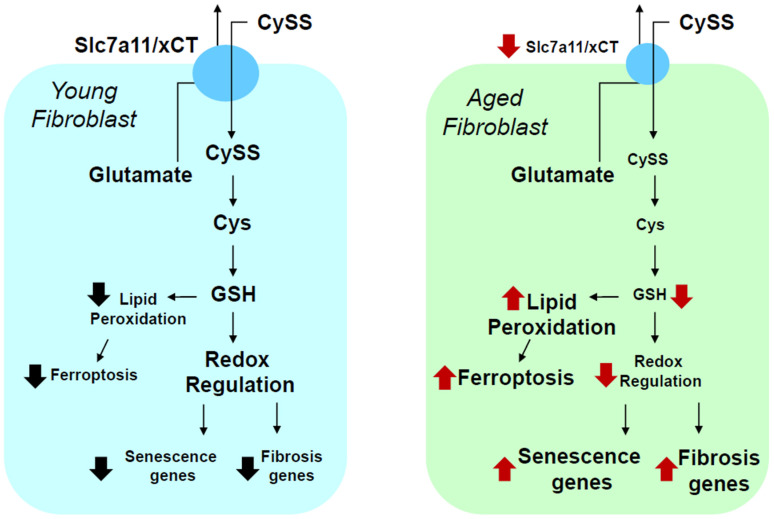
The aging lung fibroblast. In contrast to young fibroblasts (left image in ‘blue’), aging lung fibroblasts (right image in ‘green’) show decreased expression of Slc7a11, a transmembrane protein present in the plasma membrane that is a component of the xCT cystine-glutamate co-transporter. This transporter provides intracellular cystine, a rate-limiting step in the production of glutathione [125]. xCT results in decrease glutathione (GSH) levels and redox dysregulation, increased expression of profibrotic and senescence genes, and may show lipid peroxidation and ferroptosis (right image) [120].

Through a transcriptome analysis and other tests, the inability of old lung fibroblasts to reduce their oxidized environment was found to be due to a decreased expression of the solute carrier family 7, member 11 (Slc7a11), a transmembrane protein present in the plasma membrane, which is a component of the xCT cystine-glutamate co-transporter that provides intracellular cystine, a rate-limiting step in the production of glutathione [125]. More recently, it was found that a decreased expression of Slc7a11 in lung fibroblasts is associated with increased expressions of senescence markers (p21, p16, p53, and β-galactosidase), as well as pro-fibrotic markers (TGFβ, fibronectin EDA, α-smooth muscle actin, and collagen I and V). Interestingly, this phenotype was reversed by manipulating Slc7a11 expression through genetic and pharmacological interventions [120]. Specifically, the over expression of Slc7a11 or treatment with the Nrf-2 inducer, sulforaphane, increased this Slc7a11 expression while inhibiting the pro-fibrotic and senescence phenotypes of cultured aging lung fibroblasts. In contrast, the silencing of Slc7a11 or its inhibition with sulfasalazine in young fibroblasts promoted a pro-fibrotic and senescence phenotype [120] (Figure 8).

Together, these studies point to aging as a multifactorial process, which alters fibroblasts by promoting a pro-fibrotic and senescence phenotype that promotes transitional remodeling, a process that might ultimately lead to tissue disrepair after injury.

## 11. Conclusions and Research Needs

Tissue injury is characterized by the accumulation of activated fibroblasts and their production of ECMs and bioactive molecules that influence repair. While fibroblasts are clearly important for appropriate wound healing, the disruption of these events may result in unregulated fibroproliferation and the effacement of the original tissue architecture through the excessive and erratic deposition of connective tissue matrices. These events are believed to underpin the human disorders that affect the lungs, but also the kidneys (e.g., glomerulosclerosis), skin (e.g., scleroderma), heart (e.g., remodeling after myocardial infarction), and liver (e.g., cirrhosis), among other organs. These disorders and the suspected roles of fibroblasts and ECMs have been well described in recent excellent reviews [57,126,127,128,129]. In general, tissue disrepair has been estimated to contribute to 45% of all causes of death in the U.S. [130].

The relatively recent approval of two drugs capable of slowing down the decline in lung function in IPF and other progressive fibrosing pulmonary disorders suggests that the events that drive these conditions can be safely targeted in humans. However, more information is needed to develop effective interventions capable of halting, and preferably reversing, tissue damage, as well as to identify the biomarkers of disease susceptibility and therapeutic response. Accelerating the innovation in this area will require new experimental models that better resemble the human condition, a more detailed definition of the origin and identification of the phenotypic changes that fibroblasts undergo during their recruitment and activation, and the development of interventions designed to attenuate the aging fibroblast phenotype or induce the apoptosis of these cells when activated.

One key area that needs more attention relates to fibroblast-derived ECMs. Despite the early recognition that ECMs influence cell behavior through the discovery of integrins in the 1980s, interventions profiting from this knowledge have not resulted in advances in clinics. Equally disappointing has been the lack of interventions designed to inhibit profibrotic factors such as TGFβ. Uncontrolled fibroblast accumulation, excessive ECM production, and TGFβ induction represent well-known steps in the fibrogenic pathway that deserve attention.

In short, fibroblasts are mesenchymal cells necessary for carrying out wound healing after injury. However, if uncontrolled, these very cells drive tissue disrepair, fibrosis, and a loss of organ function. Targeting these cells and their downstream effects is expected to improve outcomes in the human disorders characterized by fibroproliferation.

## Data Availability

Not applicable.
